# Malay Language Translation, Cultural Adaptation and Pilot Testing of the SARC-F Questionnaire for Screening Sarcopenia Among the Malaysian Population

**DOI:** 10.21315/mjms-12-2025-912

**Published:** 2026-06-30

**Authors:** Muhammad Akmal Hakimi Mohd Aris, Mohamad Sayyidil Irfan Mohd Sufian, Haziq Zahiruddin, Wan Fatein Nabeila Wan Omar, Hazulin Mohd Radzuan, Nurul Amirah Mustapa, Wei Xin Gan, Nur Munirah Mat Salleh, Muhammad Harith Rosdi, Mohd Zulkifli Mohamad Zahir, Mohamad Suhaizi Suhaimi, Nazira Zubir, Hanifah Eydel, Nur Fazlen Zainul Abidin

**Affiliations:** 1 Kulliyyah of Medicine, International Islamic University of Malaysia, 25200 Kuantan, Pahang, Malaysia; 2 Department of Basic Medical Sciences, Kulliyyah of Medicine, International Islamic University Malaysia, 25200 Kuantan, Pahang, Malaysia; 3 Department of Physical Rehabilitation Sciences, Kulliyyah Allied Health Sciences, International Islamic University of Malaysia, 25200 Kuantan, Pahang, Malaysia; 4 Silvercare Project, ALPRO Group Head Office and Distribution Centre, Unit V1-A, Lot 45880, Jalan Techvalley 3/2, Sendayan Techvalley, 71950 Bandar Sri Sendayan, Negeri Sembilan, Malaysia; 5 Department of Library and Information Science, Kulliyyah of Information and Communication Technology, International Islamic University Malaysia, 53100 Kuala Lumpur, Malaysia; 6 IIUM Sports Medicine Clinic, International Islamic University of Malaysia, 25200 Kuantan, Pahang, Malaysia; 7 Department of Internal Medicine, Kulliyyah of Medicine, International Islamic University Malaysia, 25200 Kuantan, Pahang, Malaysia; 8 Department of Malay Language, Kulliyyah of Sustainable Tourism and Contemporary Languages, International Islamic University Malaysia Pagoh Edu Hub, KM 1, Jalan Panchor, 84600 Muar, Johor, Malaysia; 9 Department of Biology, Centre for Foundation Studies, International Islamic University Malaysia, 26300 Gambang, Pahang, Malaysia; 10 Department of English Language, Centre for Languages and Pre-University Academic Development (CELPAD), Centre for Foundation Studies, International Islamic University Malaysia, 26300 Gambang, Pahang, Malaysia; 11 Department of Psychiatry, Kulliyyah of Medicine, International Islamic University Malaysia, 25200 Kuantan, Pahang, Malaysia

**Keywords:** sarcopenia, muscular atrophy, health surveys, geriatric assessment, reliability, validity

## Abstract

**Background:**

Sarcopenia is an increasing concern among ageing populations as it contributes to frailty, falls, and reduced quality of life. Early screening is critical to prevent this disorder. The SARC-F questionnaire is known as a simple tool that can be used to screen for sarcopenia. It has been translated and validated in multiple languages. However, no validated Malay version currently exists, creating a critical gap for sarcopenia screening among Malay-speaking adults who primarily communicate in the Malay language. This study aimed to translate and culturally adapt the SARC-F questionnaire into a Malay version and to validate it preliminarily.

**Methods:**

The questionnaire was subjected to forward–backward translation, followed by expert panel evaluation (*n* = 8). Face validity was carried out among 25 Malay-speaking community-dwelling adults to assess the clarity and comprehensibility of the Malay SARC-F questionnaire using the Face Validity Index (FVI). Reliability was assessed using internal consistency (Cronbach’s α) and test–retest reliability (Intraclass Correlation Coefficient, ICC).

**Results:**

A translated Malay SARC-F was agreed after the forward translation. The backward translation of the Malay SARC-F showed minimal discrepancy from the original version. Expert panels agreed to revise certain phrases and terms to better suit the Malaysian context to optimise clarity and comprehension. Face validity was also excellent (I-FVI = 1.00; S-FVI = 1.00). Internal consistency was acceptable (Cronbach’s α = 0.78), and test–retest reliability was excellent (ICC = 0.88; 95% CI: 0.83, 0.92).

**Conclusion:**

The Malay SARC-F demonstrates preliminary evidence of reliability as a culturally appropriate screening instrument for sarcopenia among Malaysian adults. Although this study establishes its preliminary psychometric soundness, larger studies are warranted to confirm construct and criterion validity.

## Introduction

Sarcopenia, the age-related decline in skeletal muscle mass, strength, and physical performance, is currently recognised as a disease with substantial clinical implications, including increased mortality, higher hospitalisation risk, falls and fractures, adverse post-operative outcomes, cardiometabolic dysfunction, and functional decline (1–3). The Asian Working Group for Sarcopenia (AWGS) provides widely used diagnostic guidelines for sarcopenia in Asian populations, with the most recent update published in 2025, which outlines criteria based on muscle strength, muscle mass, and physical performance (4). While primary sarcopenia occurs among the geriatric population, secondary sarcopenia affects the non-elderly population associated with chronic disease, physical inactivity and other risk factors. The global prevalence of sarcopenia among people over 60 years is approximately 10% to 27%, with rates rising markedly in the oldest-old cohorts (aged over 85 years) (5). As the figure of the world’s population of people aged 60 years and older is projected to increase, sarcopenia is anticipated to cause an increase in functional dependence and healthcare demand in this population.

In Malaysia, community-based studies estimated a range of 14% to 20% of sarcopenia prevalence in people aged 60 years and older, depending on the diagnostic criteria (6). Malaysia’s demographic transition toward an older population—projected to reach approximately 15% aged 65 years and older by 2040—portends a growing burden of sarcopenia and its sequelae (7). Early identification of sarcopenia enables preventive interventions such as resistance training and improved nutrition, potentially reducing disability and healthcare expenditure.

Objective measures such as dual energy x-ray absorptiometry (DXA), bioelectrical impedance analysis (BIA), and functional tests (e.g., handgrip strength and gait speed) are diagnostically robust to diagnose sarcopenia, but these are resource- and time-intensive (8). Consequently, there is a need for brief, low-cost screening tools for sarcopenia deployable in primary care and community settings. Several self-report screening tools have been developed to facilitate early detection of sarcopenia in community and clinical settings, including the SARC-F, SARC-CalF, Ishii test, and Mini Sarcopenia Risk Assessment (MSRA) (9–12). The SARC-F questionnaire, a widely used questionnaire, is a five-item self-reported instrument assessing strength (S), assistance with walking (A), rising from a chair (R), climbing stairs (S), and falls (F). Each domain is rated on 0 to 2 scale. The cumulative score ranges from 0 to 10, with a threshold of ≥ 4 indicating a high likelihood of sarcopenia and warranting further diagnostic assessment (11, 13). Another validated screening tool is the SARC-CalF, a questionnaire combining SARC-F with calf circumference for greater sensitivity (9). On the other hand, MSRA evaluates lifestyle and clinical risk factors (12). Meanwhile, the Ishii screening test incorporates age, grip strength, and calf circumference (10).

The SARC-F’s strengths are its simplicity, feasibility, and minimal time requirement, making it highly practical for large-scale community screening and clinical use. Compared to tools such as SARC-CaIF and MSRA, the SARC-F is preferred for its ease of administration, independence from anthropometric measurements or nutritional data, and consistent cross-cultural adaptability (8, 11). However, its accurate use requires appropriate cultural and linguistic adaptation. Meta-analyses have shown that the SARC-F demonstrates high specificity (≈ 85 to 90%) but lower sensitivity (≈ 40 to 50%) for identifying sarcopenia, meaning it is better at ruling in than ruling out the condition (14–16). Over the past decade, the tool has been translated, cross-culturally adapted and validated across diverse populations globally. In Spain, it has been applied to community-dwelling older adults with good specificity for sarcopenia screening (17–19).

In addition, Portuguese studies confirmed reliability in community-dwelling older adults (20). The Greek version has been adapted for older adults and demonstrated comparable performance to other translations (21). Similarly, Turkish validation studies in geriatric outpatients reported good specificity but limited sensitivity (22). In Asia, SARC-F has also been translated into Chinese (Mandarin) and validated in both community and hospitalised cohorts, supporting its use in multiple clinical settings in China (23). Furthermore, the Korean version of SARC-F was tested among community-dwelling older adults, confirming feasibility though with modest sensitivity (24). Meanwhile, in Japan, the validation of Japan SARC-F has extended to both community residents and clinical groups such as patients with diabetes and chronic liver disease (25). The Arabic SARC-F has also been validated among community-dwelling older adults in the Middle East, demonstrating good reliability and construct validity (26, 27).

To date, no validated Malay version of the SARC-F exists. Given that the Malay language is the national language and widely spoken in Malaysia, a culturally adapted Malay SARC-F is essential to facilitate sarcopenia screening among Malay-speaking adults, particularly those with limited English proficiency. This study, therefore, aimed to translate, culturally adapt, and conduct face validity of the Malay SARC-F questionnaire. In line with established cross-cultural adaptation and psychometric evaluation protocols, the process involved forward–backward translation and cultural adaptation, harmonisation by an expert panel, and assessment of reliability through internal consistency and test–retest analysis.

## Methods

### Phase 1: Translation and Cultural Adaptation

The translation and cultural adaptation process was conducted in line with the Professional Society for Health Economics and Outcomes Research (ISPOR) guideline (28). Figure 1 shows the Flowchart of the SARC-F translation process. Written permission to translate the SARC-F was first obtained from its original author, Prof. Theodore Malmstrom (11, 13). Following established guidelines, forward translation was performed independently by three bilingual experts, consisting of certified freelance translators and translators affiliated with a professional linguistic service (28, 29). The researchers then reviewed the translated questionnaire and agreed on a single reconciled version. This agreed version was subsequently sent to another three bilingual experts for backward translation, who are an English lecturer specialising in language literacy, a Malay language lecturer specialising in Malay language and literature, and a biology lecturer experienced in translating Ministry of Education examination materials and academic texts. These linguistic experts focused on linguistic accuracy, clarity and cultural appropriateness of the translated items. Independent reconciliation of the translated versions was conducted by the core research team prior to expert panel review to produce a harmonised preliminary version. Finally, eight subject matter experts composed of geriatricians, physiotherapists, sport medicine physician, physiologist and anatomist reviewed both forward and backward-translated versions to ensure conceptual equivalence and clinical relevance of the Malay SARC-F with the original instrument.

As the SARC-F is an established instrument and no new items were developed, formal quantitative content validity assessment (e.g., content validity index) was not performed. Instead, the expert panel evaluated the translated items to ensure semantic, conceptual, and cultural equivalence with the original instrument, in accordance with recommended guidelines for cross-cultural adaptation of self-report measures (30).

### Phase 2: Face Validity

A cross-sectional pilot validation study was conducted from June 2024 to January 2025. This study complied with ethical standards as approved by the IIUM Research Ethics Committee (IREC-2025-99). Inclusion criteria were Malay-speaking adults who were literate and could communicate in Malay. Exclusion criteria included severe cognitive impairment, acute illness, or inability to complete assessments. Face validity was evaluated among 25 Malay-speaking adults. Subjects with known dementia or those with clinical changes between the tests (baseline and Week 2) were excluded. Eligible participants were adults aged 18 years and older, with no upper age limit. Trained personnel administered the Malay SARC-F in a quiet setting and recorded the participants’ demographic and clinical information (such as age, sex, comorbidities, medication, and body mass index). The subjects self-administered the finalised Malay SARC-F by reading and completing the questionnaire independently without assistance.

Next, these subjects were asked to assess the clarity and comprehensibility of each item in the Malay SARC-F. Each item was rated using a 4-point Likert scale (1 = not clear and understandable, 2 = somewhat clear and understandable, 3 = clear and understandable, 4 = very clear and understandable). Participants were also invited to provide verbal feedback regarding the wording and comprehensibility of the items. No major difficulties in understanding the translated items were reported, and no suggestions for wording modifications were raised.

The Face Validity Index for the item (I-FVI) was computed as the proportion of respondents rating an item as either 3 or 4, representing its item clarity and comprehensibility proportion (31). The Face Validity Index for scale (S-FVI) was calculated as the average of I-FVI scores for all items (S-FVI/Ave), and the proportion of items on the scale that achieved 100% raters in agreement for score 3 or 4 (S-FVI/UA) (31). An FVI ≥ 0.80 was considered acceptable, indicating that the item was sufficiently understandable and clear for the target population (31, 32). Subjects completed the Malay SARC-F questionnaire at baseline for the initial assessment and then re-answered the same questionnaire two weeks later (Week 2) to confirm stability of responses and reproducibility of the face validity findings. The administration mode at Week 2 remained the same, with no clinical change occurring between the tests.

### Phase 3: Preliminary Reliability

Internal consistency was tested with Cronbach’s α (33). Test–retest reliability was assessed in the 25 participants over a two-week interval using the Intraclass Correlation Coefficient (ICC) (two-way mixed effects model, absolute agreement, single measurement) (34).

### Data Analysis

Data analysis was conducted using IBM SPSS Statistics (version 27; IBM Corp., Armonk, NY, USA). Continuous variables are presented as mean ± standard deviation (SD), and categorical variables as frequencies (percentages). Cronbach’s α and ICC (two-way mixed, single measures) assessed reliability and were computed as 95% confidence interval. For interpretation, Cronbach’s α values ≥ 0.70, ≥ 0.80, and ≥ 0.90 were considered acceptable, good, and excellent, respectively (33). Meanwhile, ICC values < 0.50, 0.50 to 0.75, 0.75 to 0.90 and > 0.90 indicate poor, moderate, good, and excellent reliability (34).

## Results

### Phase 1: Translation and Cultural Adaptation

Table 1 shows the a) original and b) finalised Malay SARC-F questionnaire. During the expert panel review, a few phrases with the same meaning in the pre-harmonised Malay version of the SARC-F questionnaire (i.e., “*berapakah kesukaran*,” “*berapa banyak kesukaran*,” “*berapa anggaran kesukaran*,” and “*berapa tahap kesukaran*”) were suggested to be used, and after thorough discussion, the phrase “*berapakah kesukaran*” was selected to avoid unnecessary wording while keeping the question clear and comprehensible.

For the first component, the usage of pound was changed to kilogram (kg) to suit the usage metric unit of the local community as people in Malaysia are used to the kg unit instead of pound. The original measurement of 10 pounds was initially converted to 4.5 kg, which is the direct comparative value of the unit. To optimise cultural face validity and ease of use, 10 pounds (~ 4.5 kg) was operationalised as 5 kg, utilising a 5 kg rice pack as a culturally familiar weight anchor.

During pre-testing of the questionnaire, most of the respondents had no difficulty in understanding all components clearly, and most of them were able to rate or give a clear score. However, there was a limiting factor to the original questionnaire that was identified, which is the inability to differentiate the causes that lead to difficulty performing the actions. As the questions for all components only ask about the general difficulty, it was not enough to identify the different causes like pain, weakness and breathlessness. Although this limitation is identified, we do not change or add any content to the questionnaire or assess content validity, as it was not the intention of the study conducted.

### Phase 2: Face Validity

A total of 25 Malay-speaking adults participated in this study. Participants’ ages ranged from 29 to 88 years, with a mean of 55.2 ± 14.6 years (Table 2). Approximately two-thirds of the participants were female, and the majority were married. Hypertension and diabetes were the most commonly reported comorbidities. Among those who were employed, teaching was the most common occupation.

All five items of the SARC-F questionnaire were rated 3 (clear and comprehensible) or 4 (very clear and comprehensible) by all respondents (Table 3). The FVI values for each item exceeded the acceptable threshold of 0.80, indicating excellent face validity.

### Phase 3: Preliminary Reliability

All 25 subjects reassessed the Malay SARC-F at Week 2. The internal consistency of the Malay version of the SARC-F questionnaire was found to be acceptable, with a Cronbach’s alpha value of 0.78. This suggests a good level of consistency among the questionnaire items. Additionally, test–retest reliability showed excellent reliability with an ICC of 0.88 (95% CI: 0.83, 0.92), indicating strong agreement between repeated measurements.

## Discussion

This study reports the translation, cultural adaptation and pilot psychometric testing of Malay SARC-F among Malay-speaking adults. The Malay SARC-F demonstrated strong measurement properties; linguistic equivalence, clarity and short-term score stability, supporting its suitability as a simple screening tool for sarcopenia in community settings. The instruments are carefully translated by certified lingual experts, and they have been specifically tailored to make sure the items used are relevant. This is proved by a strong Face Validity Index (FVI = 1.00), indicating good comprehension among target users. Reliability indices, including Cronbach’s α of 0.78 and ICC of 0.88, indicate acceptable internal consistency and good temporal reproducibility for the Malay SARC-F. However, the interpretation of Cronbach’s αshould be made with caution given the relatively small sample size (*n* = 25), which may produce unstable estimates. In addition, the SARC-F comprises five items assessing related but distinct aspects of sarcopenia risk, including strength, mobility, and falls. As such, the scale may reflect a multidimensional construct, and very high internal consistency is not necessarily expected. The observed reliability estimates are generally comparable to those reported in other SARC-F validation studies, although direct comparisons should be interpreted cautiously due to differences in study populations and sample sizes.

Our findings are consistent with previous translation and validation efforts conducted in other languages, including Japanese, Turkish, and Arabic (22, 25–27). Similar to these studies, the Malay version demonstrated the importance of cultural adaptation to preserve the questionnaire’s performance across diverse linguistic and sociocultural contexts. For example, the forward–backward translation process, expert harmonisation, and cognitive pretesting used in this study likely contributed to ensuring semantic clarity and cultural appropriateness. To strengthen the diagnostic accuracy of the Malay SARC-F, the questionnaire requires a larger criterion validity study, comparing it against the standard sarcopenia diagnostic criteria and assessing for sensitivity and specificity. Importantly, the results highlight the value of rigorous translation methodology, as direct linguistic conversion alone is insufficient when transferring screening tools across populations with differing cultural norms, languages, and health literacy levels.

The present findings also have practical implications. Given Malaysia’s rapidly ageing population and the increasing burden of age-related conditions such as sarcopenia, there is an urgent need for simple, low-cost screening methods that can be applied in primary care and community health settings. The Malay SARC-F, being brief, self-reported, and noninvasive, meets these criteria. In resource-limited contexts, where access to advanced diagnostic modalities such as DXA or BIA may be restricted, the Malay SARC-F offers a pragmatic first-line screening option. Early identification of individuals at risk can facilitate timely referral, preventive interventions, and targeted management strategies aimed at reducing frailty, falls, and loss of independence.

Despite these strengths, several limitations must be acknowledged. First, the use of purposive sampling may limit the generalisability of findings across Malaysia’s diverse ethnic and cultural groups. Second, the conversion of the original imperial unit (10 pounds) to a rounded metric equivalent (5 kg) may have introduced minor differences in respondents’ interpretation. Third, the SARC-F cannot distinguish difficulties caused by muscle weakness from those due to pain or comorbidities, potentially reducing diagnostic specificity. In addition, although participants provided verbal feedback on item clarity, formal cognitive debriefing interviews were not conducted, which may limit the depth of insight into respondents’ interpretation of the questionnaire items. Lastly, the small sample size (*n* = 25), single-site recruitment, and lack of criterion validity assessment limit the robustness and generalisability of the psychometric findings. Factor analysis was not performed due to the small sample size (*n* = 25), which is below commonly recommended thresholds for stable factor extraction. Furthermore, the SARC-F is a brief screening instrument consisting of only five items, and the present study aimed to perform translation and preliminary validation rather than scale development. Future larger, multi-site studies are recommended to strengthen the evidence for the Malay SARC-F.

Future studies should therefore extend validation efforts to larger, more diverse samples across different Malaysian states and ethnic groups, ensuring the tool’s applicability in the broader national context. More importantly, the Malay SARC-F questionnaire should be validated for criterion validity against the diagnostic criteria of sarcopenia. Additional research could also explore the incorporation of culturally relevant measurement anchors, further harmonisation of metric unit equivalents, and modifications to enhance discriminative ability between sarcopenia-related weakness and functional impairments arising from pain or other conditions. Moreover, prospective studies assessing predictive validity—such as the ability of the Malay SARC-F to forecast adverse outcomes like falls, hospitalisation, or mortality—would provide valuable evidence for its utility in clinical practice and public health screening programmes.

This study demonstrates preliminary linguistic validity and reliability of the Malay SARC-F as a screening tool for sarcopenia; however, construct and criterion validity remain to be established. The findings highlight the importance of rigorous cross-cultural adaptation when applying health measurement instruments in different linguistic and cultural contexts. The availability of a validated Malay version may facilitate sarcopenia screening and raise awareness of age-related muscle decline in Malaysia’s ageing population.

## Conclusion

The Malay SARC-F demonstrates preliminary evidence of reliability as a culturally appropriate screening instrument for sarcopenia among Malay-speaking adults. Its simplicity supports potential application in community and primary care settings. However, these findings should be interpreted with caution due to the small, single-site sample. Future studies involving larger, multi-ethnic populations and evaluation against the AWGS 2025 diagnostic criteria are needed to establish its reliability and clinical applicability further.

## Figures and Tables

**Figure 1:**
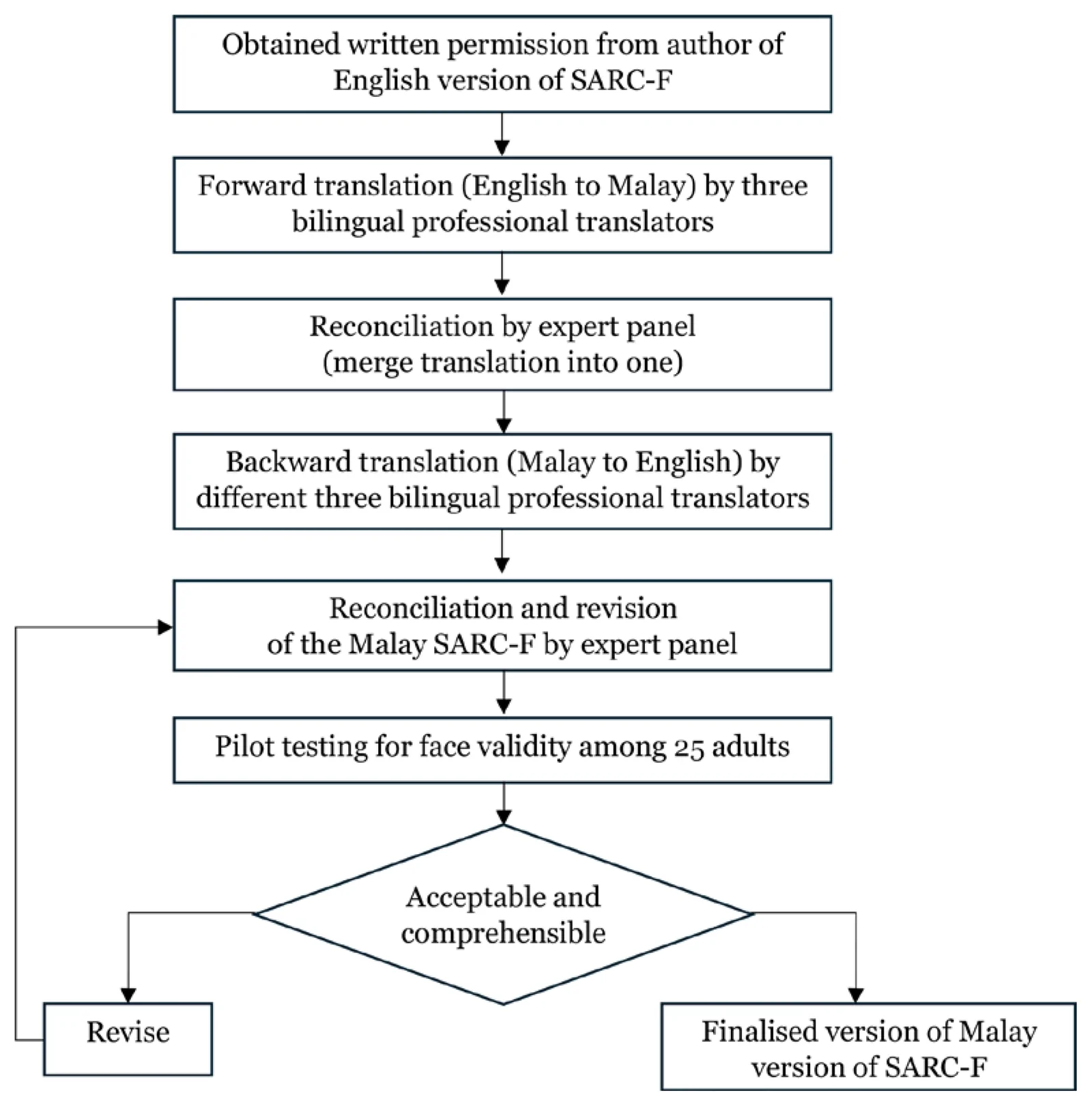
Flowchart of Malay SARC-F translation process.

**Table 1:** a) Original SARC-F for screening sarcopenia (10), and b) Finalised Malay SARC-F for screening sarcopenia in the Malaysian population.

a) Original SARC-F			b) Finalised Malay SARC-F		
Component	Question	Scoring	*Komponen*	*Soalan*	*Pemarkahan*
Strength	How much difficulty do you have in lifting and carrying 10 pounds?	None = 0 Some = 1 A lot or unable = 2	*Kekuatan*	*Berapakah kesukaran yang anda alami semasa mengangkat dan membawa sekampit beras seberat 5 kg?*	*Tiada = 0* *Sedikit = 1* *Banyak atau tidak mampu = 2*
Assistance in walking	How much difficulty do you have walking across a room?	None = 0 Some = 1 A lot, use aids, or unable= 2	*Bantuan dalam berjalan*	*Berapakah kesukaran yang anda alami semasa berjalan dalam sebuah bilik dari satu sudut ke sudut yang lain?*	*Tiada = 0* *Sedikit = 1* *Banyak, menggunakan alat bantu, atau tidak mampu = 2*
Rise from a chair	How much difficulty do you have transferring from a chair or bed?	None = 0 Some = 1 A lot or unable without help= 2	*Bangun dari kerusi*	*Berapakah kesukaran yang anda alami semasa bangun dari kerusi atau katil?*	*Tiada = 0* *Sedikit = 1* *Banyak atau tidak mampu tanpa bantuan = 2*
Climb stairs	How much difficulty do you have in climbing a flight of 10 stairs?	None = 0 Some = 1 A lot or unable = 2	*Menaiki tangga*	*Berapakah kesukaran yang anda alami untuk menaiki 10 anak tangga?*	*Tiada = 0* *Sedikit = 1* *Banyak atau tidak mampu = 2*
Falls	How many times have you fallen in the past year?	None = 0 1–3 falls = 1 4 or more falls = 2	*Jatuh*	*Berapakah kekerapan anda jatuh dalam tempoh setahun yang lalu?*	*Tiada = 0* *1–3 kali = 1* *4 kali atau lebih = 2*

**Table 2:** Sociodemographic of the subjects for the face validity of Malay SARC-F.

Variable	*n* (%)	Mean ± SD	Range
Age (year)	-	55.2 ± 14.6	29 to 88
Female	17 (68)	-	-
Malay	24 (96)	-	-
Islam	24 (96)	-	-
Married	20 (80)	-	-
Tertiary education and above	23 (92)	-	-
Full time employed	12 (48)	-	-
Retiree/unemployed	13 (52)	-	-
Has comorbidities	17 (68)	-	-
Smoking	4 (16)	-	-
Consume alcohol	1 (4)	-	-

*n* (%) = frequency (percentage); SD = standard deviation.

**Table 3:** FVI for the Malay SARC-F.

Item	Raters in agreement, *n* (%)	I-FVI	Universal agreement
Q1	25 (100)	1.0	1.0
Q2	25 (100)	1.0	1.0
Q3	25 (100)	1.0	1.0
Q4	25 (100)	1.0	1.0
Q5	25 (100)	1.0	1.0
S-FVI/Ave	-	1.0	-
S-FVI/UA	-	-	1.0

FVI = Face Validity Index; I-FVI = Item level Face Validity Index; Raters in agreement = number of subjects (out of 25) who rated the item as 3 (clear and understandable) or 4 (very clear and understandable); S-FVI/Ave = Scale-level Face Validity Index based on the average method; S-FVI/UA = Scale-level Face Validity Index based on the universal agreement method; Universal agreement of score 1= item rated as score 3(clear and understandable) or 4 (very clear and understandable) by 100% subjects.
